# Unilateral Dual‐Plane Puncture Percutaneous Vertebroplasty Reduces Re‐Collapse in Osteoporotic Vertebral Compression Fractures by Advancing Cement Delivery

**DOI:** 10.1111/os.70004

**Published:** 2025-04-02

**Authors:** Huo‐Liang Zheng, Chang‐hai Liu, Lei‐Sheng Jiang, Xin‐Feng Zheng, Sheng‐Dan Jiang

**Affiliations:** ^1^ Department of Clinic of Spine Center Xinhua Hospital, Shanghai Jiaotong University School of Medicine Shanghai China; ^2^ Chongming Hospital Affiliated to Shanghai University of Medicine and Health Sciences Shanghai China

**Keywords:** osteoporotic vertebral compression fractures, percutaneous vertebroplasty, re‐collapse, unilateral dual‐plane puncture

## Abstract

**Purpose:**

Evaluate the efficacy of a novel unilateral dual‐plane puncture technique in improving bone cement distribution and reducing vertebral re‐collapse following percutaneous vertebroplasty (PVP) for osteoporotic vertebral compression fractures (OVCFs). By introducing the novel unilateral dual‐plane puncture technique, this study aims to improve cement distribution, reduce the incidence of re‐collapse, and enhance long‐term clinical outcomes for patients suffering from OVCFs.

**Methods:**

This is a randomized trial conducted from April 2021 to December 2022, enrolling 145 patients diagnosed with OVCFs. Patients were allocated into either traditional or unilateral dual‐plane puncture groups. Bone cement distribution, vertebral height, and segmental kyphotic angle were measured through postoperative x‐ray, while clinical outcomes were evaluated using the Visual Analog Scale (VAS) and the Oswestry Disability Index (ODI). Statistical analysis was performed using the Mann–Whitney *U* test and independent samples *t* test for continuous variables, and chi‐square or Fisher's exact test for categorical variables.

**Results:**

The unilateral dual‐plane puncture technique notably augmented bone cement contact with both superior and inferior endplates compared to conventional methods, achieving rates of 64.86% versus 40.85% (*p* < 0.001). This contributed to a significant reduction in the incidence of vertebral re‐collapse within the first year post‐operation: 18.92% in the unilateral dual‐plane group as opposed to 42.25% in the traditional group (*p* < 0.001). Furthermore, the unilateral dual‐plane group exhibited markedly superior long‐term efficacy, evidenced by mean VAS and ODI scores of 1.26 and 28.58, respectively, in comparison to 2.03 and 32.45 in the traditional group.

**Conclusions:**

The unilateral dual‐plane puncture technique advances bone cement distribution within the vertebra, thereby reducing the risk of vertebral re‐collapse following PVP surgery and improving long‐term clinical outcomes for patients with OVCFs.

## Graphical abstract

Trajectory of the unilateral dual‐plane puncture. The sagittal CT scan after surgery clearly shows the puncture trajectory of the unilateral dual‐plane puncture. The bone cement simultaneously contacted both the upper and lower end plates.

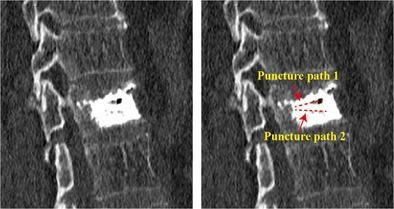

## 1 Introduction

With the aging population and increasing emphasis on quality of life, osteoporosis is gradually becoming a societal concern for the elderly [1, 2, 3]. Fragility fractures resulting from osteoporosis represent a severe consequence, as reduced bone mass, decreased bone strength, and increased bone fragility make even minor trauma in daily activities capable of causing brittle fractures [4, 5, 6]. Such fractures often manifest as complete fractures, with osteoporotic vertebral compression fractures (OVCFs) being one of the most prevalent types [7, 8].

The incidence of osteoporotic vertebral compression fractures is expected to increase with the intensification of population aging [9]. Treatment for these fractures includes conservative and surgical approaches. Conservative treatments involve bed rest, orthopedic bracing, and similar measures [10, 11, 12]. Common surgical interventions include fracture reduction and internal fixation, as well as percutaneous vertebral augmentation procedures [13, 14]. Percutaneous vertebral augmentation comprises percutaneous vertebroplasty (PVP) and percutaneous kyphoplasty (PKP), both of which effectively address osteoporotic vertebral compression fractures through minimally invasive procedures, alleviating pain, restoring vertebral height, and enhancing spinal stability [15, 16, 17, 18, 19].

However, a phenomenon of postoperative vertebral height loss has been observed in both PVP and PKP, which can lead to a return to preoperative states, exacerbating pain and causing spinal deformities, sometimes necessitating revision surgery. Lin et al. referred to the anterior height loss of vertebrae after PVP as “re‐fracture” while Heo et al. described the post‐PVP vertebral height loss as “re‐collapse” [20, 21]. Some studies suggest a close association between the distribution, morphology, and injection volume of bone cement and post‐PVP vertebral re‐collapse. The morphology and distribution of bone cement are believed to be associated with postoperative vertebral re‐collapse [22]. Li et al. suggest that the distance between the bone cement and the upper endplate of the injured vertebra can more accurately reflect the dispersion of the cement [23]. A large distance increases the risk of postoperative height loss, and vertebral re‐collapse mainly occurs where cement is not filled.

Therefore, we introduced a novel unilateral dual‐plane PVP puncture technique aimed to (i) Evaluate the efficacy of a novel unilateral dual‐plane puncture technique in improving bone cement distribution within the vertebral body; (ii) Investigate the impact of this technique on reducing the incidence of vertebral re‐collapse following PVP for OVCFs; (iii) Assess the long‐term clinical outcomes, including pain relief and functional recovery, in patients treated with the unilateral dual‐plane puncture technique compared to traditional PVP methods.

## 2 Methods

### 2.1 Population

Power size calculation was performed to ensure the study had sufficient statistical power to detect significant differences between the groups. The calculated sample size was based on the primary outcomes of re‐collapse rates. Under the assumption of the expected collapse rate, each group (traditional group and unilateral dual‐plane puncture group) was estimated to require at least 47 patients to achieve sufficient statistical power.

This study included patients who underwent PVP surgery at our hospital from April 2021 to December 2022. This study employed a random allocation method, where patients were assigned to either the traditional group or the unilateral dual‐plane puncture group using random numbers. The randomization process was conducted by an independent researcher. All enrolled patients underwent clinical assessment. Inclusion criteria were as follows: (1) Thoracolumbar vertebral compression fractures below T8 without additional fractures; (2) Males aged ≥ 55 years and postmenopausal females; (3) Single‐segment OVCF and preoperative radiological examinations confirming fresh OVCF showing high signal on T2‐weighted fat‐suppressed MRI images; (4) Lumbar spine bone mineral density measured by dual‐energy x‐ray absorptiometry (DXA), with T value < −2.5 standard deviations (SD). Exclusion criteria included: (1)Spinal infection, thoracolumbar neoplastic fractures, and severe trauma occurring before enrollment; (2) Chronic corticosteroid use. (3) Other bone metabolism disorders such asosteomalacia. (4) Vertebral body posterior wall injury. A total of 189 patients were included in this study, with 145 patients completing the full follow‐up. This study was approved by the institutional review board of Xinhua Hospital, affiliated with Shanghai Jiao Tong University School of Medicine(XHEC‐QT‐2021‐086). All participants received both written and verbal information before providing written consent, and the study was conducted in accordance with the principles of the Helsinki Declaration.

### 2.2 Intervention

**FIGURE 1:**
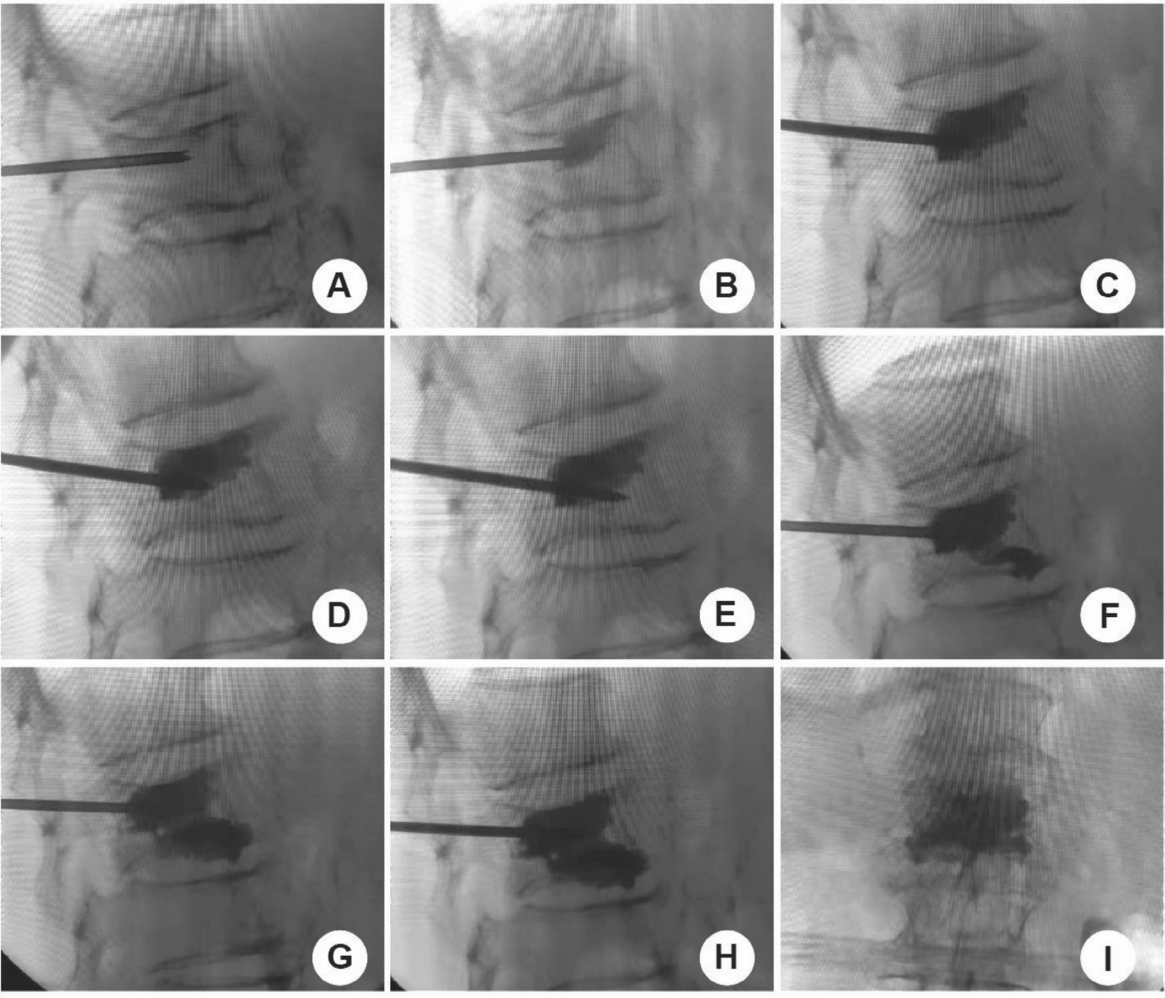
Unilateral dual‐plane puncture technique under C‐arm imaging. (A) The puncture needle is directed close to the upper endplate, reaching the anterior middle third of the vertebral body. (B) Injection of an appropriate amount of bone cement. (C) Withdrawal of the puncture needle. (D) Adjustment of the puncture direction towards the lower endplate. (E) The puncture needle reaches the mid‐lower part of the vertebral body. (F) Injection of an appropriate amount of bone cement. (G, H) While retracting the puncture needle, inject bone cement simultaneously. (I) Postoperative spine anteroposterior x‐ray acquired by C‐arm machine.

**FIGURE 2:**
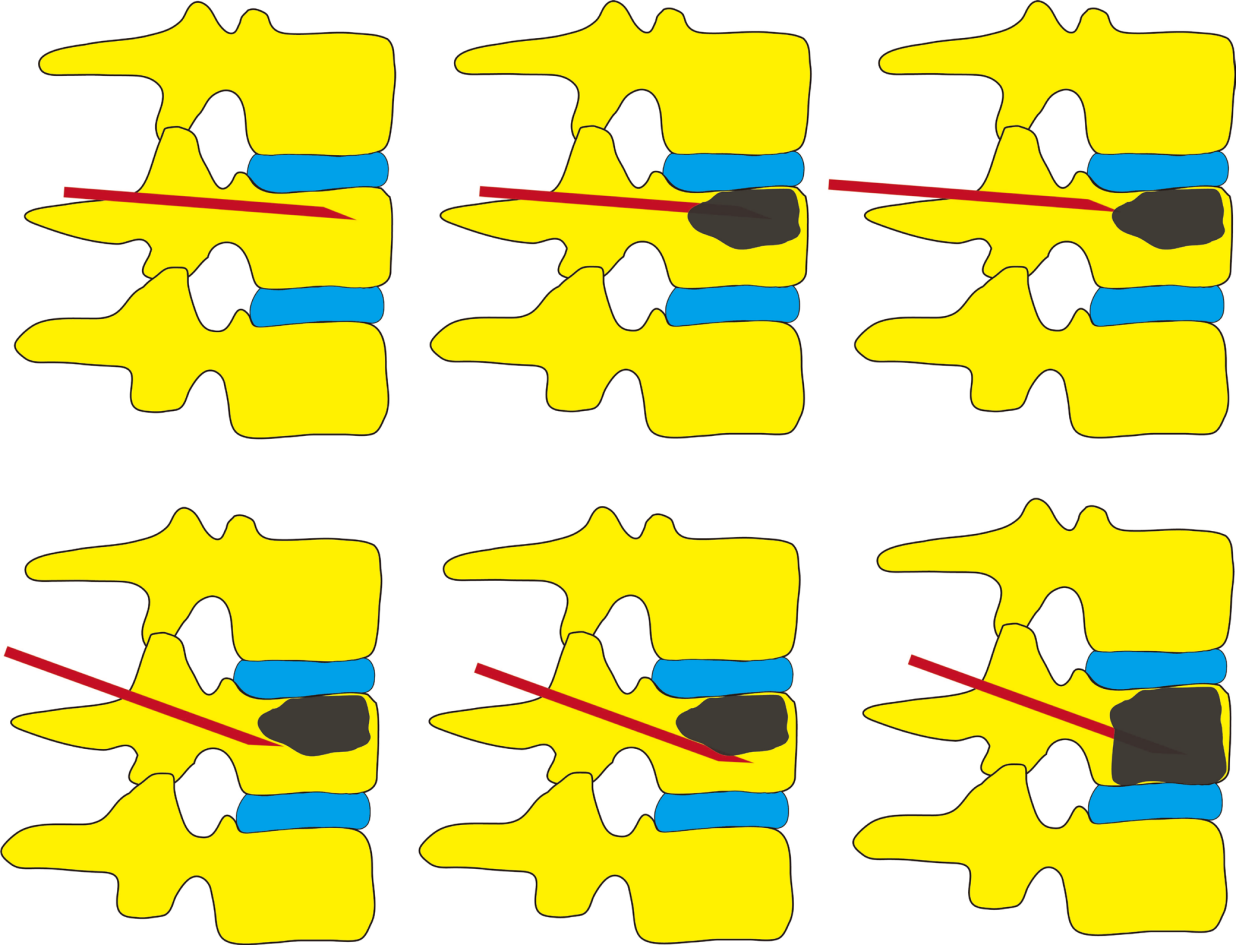
Unilateral dual‐plane vertebral augmentation surgery schematic.

**FIGURE 3:**
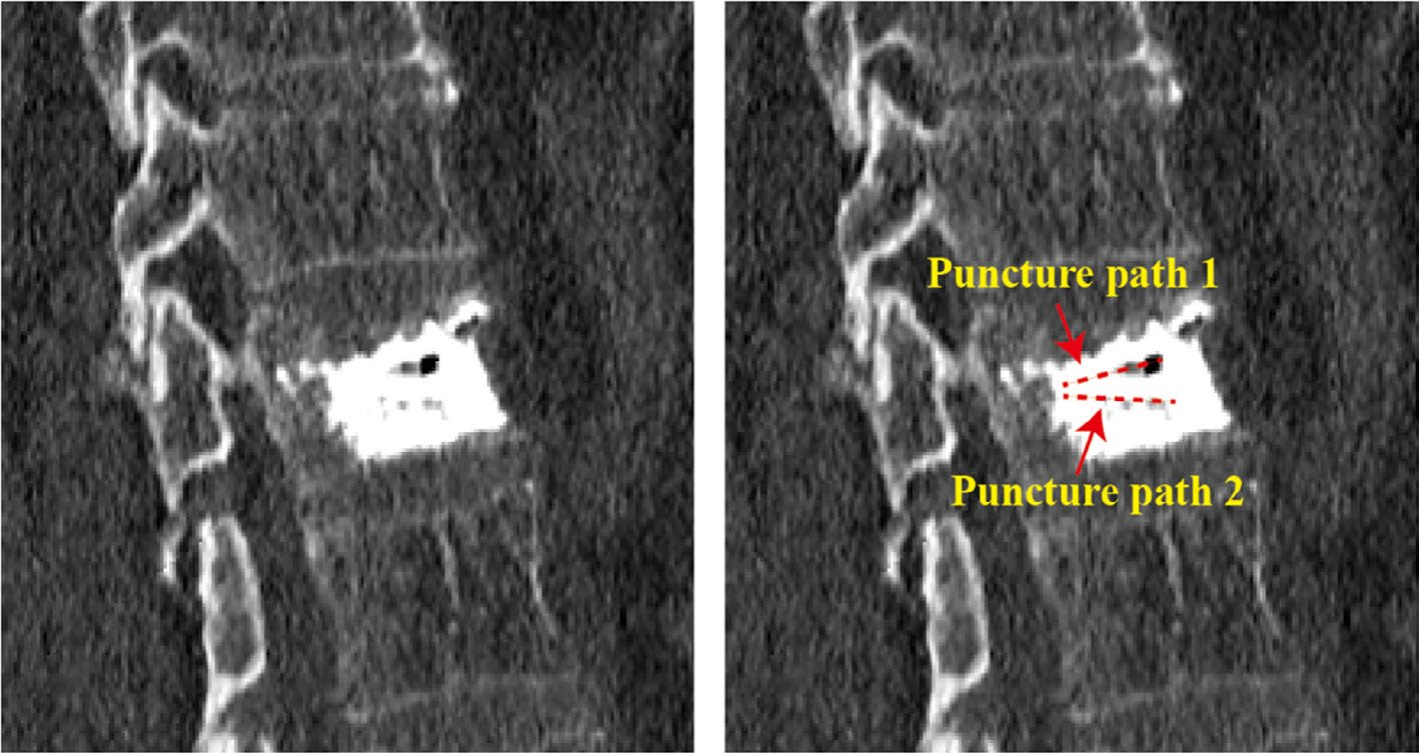
Postoperative CT sagittal images of unilateral dual‐plane puncture showing the original puncture path and the adjusted puncture path.

All PVP surgeries were performed by the same surgeon (Jiang SD). Patients underwent either traditional puncture or unilateral dual‐plane puncture. The patient was positioned prone, and the location of the fracture was determined utilizing C‐arm fluoroscopy. The surgical procedure was performed under local anesthesia (1% lidocaine). Guided by x‐ray, the needle was inserted at either the 10 o'clock position on the left pedicle or the 2 o'clock position on the right pedicle. In the traditional puncture approach, the needle was guided into the vertebral body under x‐ray guidance, and an appropriate amount of bone cement was injected without readjusting the needle direction. During the unilateral dual‐plane PVP puncture, the needle was slightly tilted towards the upper endplate of the vertebra. When the needle reached the anterior one‐third of the vertebral body, bone cement was injected under monitoring by the C‐arm machine. Subsequently, the puncture needle was withdrawn to the posterior edge of the vertebra, and the angle of the needle was adjusted to tilt slightly towards the lower endplate of the vertebra. After reaching the anterior one‐third of the vertebral body, an appropriate amount of bone cement was injected. It can be observed that the bone cement made adequate contact with the upper and lower endplates of the vertebrae (Figure 1). The schematic diagram of the puncture method is shown in Figure 2. After the first injection, the puncture needle is withdrawn and its direction adjusted, followed by a second injection of bone cement. Postoperative CT revealed the trajectory of the puncture (Figure 3). Analgesics were administered orally only on the day of surgery.

### 2.3 Imaging

CT and MRI scans were performed preoperatively to accurately determine the location of the fracture and the extent of vertebral collapse, providing support for the surgery. x‐ray examinations were conducted preoperatively, at 1 day post‐surgery, 1 month post‐surgery, 3 months post‐surgery, and 1 year post‐surgery to measure vertebral height, local kyphotic angle (LKA), lumbar lordosis angle (LL) and thoracolumbar kyphosis angle (TLK). Local kyphotic angle was defined as the angle between the upper endplate line and the lower endplate of the fractured vertebra. The distribution of bone cement was categorized as Full Endplate Contact, Partial Endplate Contact, and No Endplate Contact. Full Endplate Contact indicates that the bone cement simultaneously contacts both the superior and inferior endplates of the injured vertebra. Partial Endplate Contact indicates that the bone cement contacts either the superior or inferior endplate of the injured vertebra. No Endplate Contact indicates that the bone cement does not contact either the superior or inferior endplate of the injured vertebra. Re‐collapse is defined as x‐ray imaging at the last follow‐up visit showing a decrease in anterior vertebral body height of more than 1.0 mm and an increase in the kyphotic angle of more than 3° compared to day 1 after surgery.

### 2.4 Clinical Assessment

Back pain severity changes were assessed using the Visual Analog Scale (VAS), while the Oswestry Disability Index (ODI) was employed to evaluate patients' capacity for daily activities. Patients were assessed preoperatively, 1 day postoperatively, 1 month postoperatively, 3 months postoperatively, and 1 year postoperatively.

### 2.5 Statistical Analysis

Statistical analysis was performed using IBM SPSS Statistics 26 (USA). Continuous variables were presented as mean ± standard deviation, and normality was assessed using the Shapiro–Wilk test to ensure appropriate analysis selection. For non‐normally distributed continuous variables, the Mann–Whitney *U* test was used for comparisons between the two groups. For normally distributed variables, the independent samples t‐test was conducted. Categorical variables were analyzed using the chi‐square test, and in cases where expected frequencies were less than 5, Fisher's exact test was employed to maintain result accuracy. All statistical tests were two‐tailed, and a significance level of *p* < 0.05 was considered statistically significant.

## 3 Results

### 3.1 General Characteristics of the Patients

These patients received PVP treatment at Xinhua Hospital, affiliated with Shanghai Jiao Tong University School of Medicine, from November 2021 to December 2022. Among them, 145 patients had complete information, with a mean follow‐up time of 14.8 months (range: 12–18 months). The traditional PVP puncture group (*n* = 71) comprised 55 females and 16 males, with a mean age of 71.32 years. The unilateral double‐plane PVP puncture group (*n* = 74) comprised 60 females and 14 males, with a mean age of 73.43 years. There was no significant difference in bone mineral density between the two groups (*p* = 0.66). Preexisting medical conditions of patients included hypertension (52 patients), diabetes (13 patients), and coronary atherosclerotic heart disease (13 patients) (Table 1). There were no significant differences between the two groups in terms of preoperative anterior vertebral height (AVH), LKA, LL, and TLK (Table 2). Also, there were no differences in preoperative VAS and ODI scores between the two groups (Table 2).

**TABLE 1:** Descriptive statistics of the subjects in the study (x ± s, *n* = 145).

Characteristics	t‐PVP (*n* = 71)	ud‐PVP (*n* = 74)	*p*
Age at surgery (years)	71.32 ± 6.53	73.43 ± 6.81	0.07^U^
Gender			
Female	55 (77.46%)	60 (81.08%)	0.68^F^
Male	16 (22.54%)	14 (18.92%)	
BMI (kg/m^2^)	24.49 ± 4.45	23.62 ± 3.98	0.22^T^
Hypertension	23 (32.39%)	29 (39.19%)	0.49^F^
Diabetes	7 (9.86%)	6 (8.11%)	0.78^F^
CHD	5 (7.04%)	8 (10.81%)	0.56^F^
BMD (T‐score)	−3.26 ± 0.45	−3.29 ± 0.45	0.66^U^
Fracture site			
T9	1 (1.41%)	2 (2.7%)	0.40^C^
T10	6 (8.45%)	2 (2.7%)	
T11	9 (12.68%)	6 (8.11%)	
T12	14 (19.72%)	20 (27.03%)	
L1	23 (32.39%)	19 (25.68%)	
L2	11 (15.49%)	14 (18.92%)	
L3	5 (7.04%)	4 (5.41%)	
L4	1 (1.41%)	6 (8.11%)	
L5	1 (1.41%)	1 (1.35%)	
Follow‐up time (months)	15.08 ± 1.96	14.57 ± 1.89	0.11^U^

*Note:* The symbol ‘U’ indicates the data were analyzed by the unpaired Mann–Whitney *U* test; The symbol ‘T’ indicates the data were analyzed by the unpaired *t* test; The symbol ‘F’ indicates the data were analyzed by the Fisher's exact test; The symbol ‘C’ indicates the data were analyzed by the Chi‐square test.

Abbreviations: BMI, body mass index; CHD, coronary artery atherosclerotic heart disease; t‐PVP, traditional puncture percutaneous vertebroplasty; ud‐PVP, unilateral dual‐plane puncture percutaneous vertebroplasty.

**TABLE 2:** Comparisons of preoperative parameters between traditional puncture PVP and unilateral dual‐plane puncture PVP (x ± s, *n* = 145).

Parameters	t‐PVP (*n* = 71)	ud‐PVP (*n* = 74)	*p*
VAS	7.45 ± 1.24	7.35 ± 1.24	0.63^T^
ODI	70.69 ± 9.91	70.0 ± 11.1	0.63^U^
Local kyphotic angle (LKA)	14.74 ± 7.89	14.97 ± 7.93	0.78^U^
Anterior vertebral height (AVH)	15.87 ± 4.34	15.51 ± 4.20	0.69^U^
posterior vertebral height (PVH)	23.74 ± 6.42	23.12 ± 6.07	0.67 ^U^
AVH/PVH	1.50 ± 0.03	1.50 ± 0.07	0.09 ^U^
Lordosis angle (LL)	41.63 ± 12.58	43.45 ± 13.7	0.41^T^
Thoracolumbar kyphosis angle (TLK)	17.17 ± 8.49	16.10 ± 7.17	0.40^T^

*Note:* The symbol ‘U’ indicates the data were analyzed by the unpaired Mann–Whitney *U* test; The symbol ‘T’ indicates the data were analyzed by the unpaired *t* test.

Abbreviations: ODI, the Oswestry Disability Index; t‐PVP, traditional puncture percutaneous vertebroplasty; ud‐PVP, unilateral dual‐plane puncture percutaneous vertebroplasty; VAS, Visual Analog Scale.

### 3.2 Bone Cement Distribution Pattern

In the unilateral dual‐plane puncture group, the needle was guided through the unilateral pedicle, supplemented by retrograde manipulation to adjust the puncture angle, with the aim of maximizing simultaneous contact between the bone cement and the superior and inferior endplates. Postoperative x‐rays were employed to assess the sagittal distribution of the bone cement within the vertebral body. Consistent with our expectations, in the traditional puncture group, 29 cases (40.85%) exhibited contact between the bone cement and the superior and inferior endplates. Notably, this proportion significantly increased to 48 cases (64.86%) in the modified puncture group, surpassing that of the traditional group. Within the traditional cohort, contact between the bone cement and only one endplate was observed in 35 instances (49.30%), while failure to engage both endplates was noted in 7 cases (9.85%), proportions notably higher than those observed in the modified group, which recorded 24 cases (32.43%) and 2 cases (2.7%), respectively (Table 3). Notably, although the mean usage of bone cement in the unilateral dual‐plane group was higher than that in the traditional group, there was no significant statistical difference between the two groups in terms of the amount of bone cement used and the rate of bone cement leakage (Table 3).

**TABLE 3:** Comparisons of postoperative parameters between traditional puncture PVP and unilateral dual‐plane puncture PVP (x ± s, *n* = 145).

Parameters	t‐PVP (*n* = 71)	ud‐PVP (*n* = 74)	*p*
Bone cement volume (mL)	5.83 ± 1.43	5.89 ± 1.47	0.78 ^T^
Bone cement distribution			
Full endplate contact	29 (40.85%)	48 (64.86%)	0.01^C^
Partial endplate contact	35 (49.30%)	24 (32.43%)	
No endplate contact	7 (9.85%)	2 (2.70%)	
Bone cement leakage	28 (39.44%)	37 (50%)	0.24^F^
LKA	11.25 ± 6.13	11.79 ± 6.46	0.71^U^
AVH	17.06 ± 3.59	17.18 ± 3.52	0.84^T^
LL	43.79 ± 12.45	45.35 ± 13.46	0.47^T^
TLK	14.52 ± 8.68	12.91 ± 7.55	0.49^U^

*Note:* The symbol ‘U’ indicates the data were analyzed by the unpaired Mann–Whitney *U* test; The symbol ‘T’ indicates the data were analyzed by the unpaired *t* test; The symbol ‘F’ indicates the data were analyzed by the Fisher's exact test; The symbol ‘C’ indicates the data were analyzed by the Chi‐square test.

Abbreviations: t‐PVP, traditional puncture percutaneous vertebroplasty; ud‐PVP, unilateral dual‐plane puncture percutaneous vertebroplasty.

### 3.3 Clinical Outcomes

**FIGURE 4:**
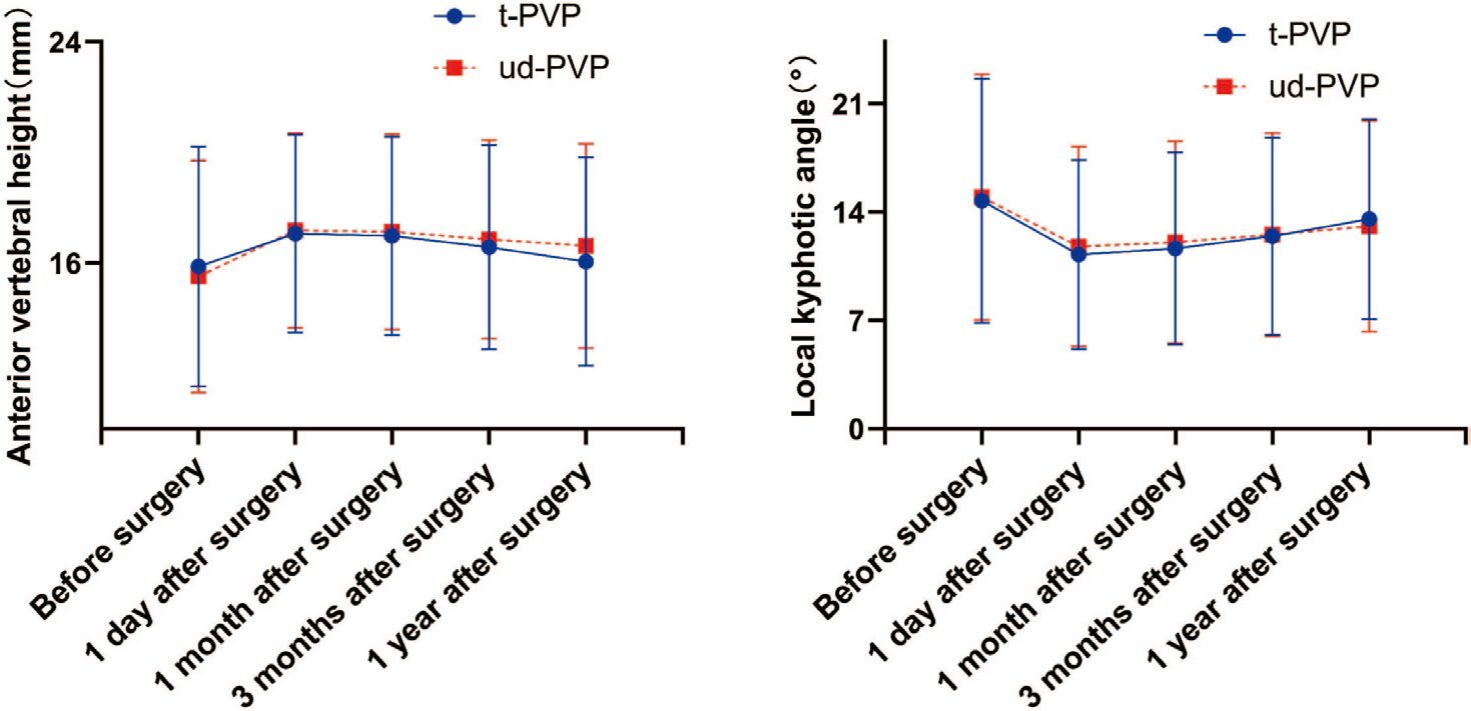
Anterior vertebral body height and local kyphotic angle of the augmented vertebra during pre‐ and postoperative follow‐up.

Although there were no significant differences in LKA, AVH, LL, and TLK between the two groups at 1 year postoperatively (Figure 4), the changes in AVH and LKA from postoperative to 1 year postoperatively were greater in the traditional group than in the unilateral dual‐plane puncture group (Table 4). Furthermore, clinical efficacy was assessed using the Visual Analog Scale (VAS) and Oswestry Disability Index (ODI). There were no significant differences in preoperative VAS and ODI scores between the two groups. On the first postoperative day, patients in both the traditional and unilateral dual‐plane puncture groups experienced significant improvement in pain, with no significant difference between the groups in terms of improvement magnitude. During the 3‐month follow‐up period, clinical efficacy was comparable between the two groups. However, at 1 year postoperatively, patients in the unilateral dual‐plane puncture group exhibited a statistically significant difference in VAS scores, with a mean of 1.26 compared to 2.03 in the traditional group. In terms of ODI scores, patients in the unilateral dual‐plane group achieved an average score of 28.58, which was superior to the traditional group's score of 32.45 (Table 5).

**TABLE 4:** Radiological parameters at 1‐year postoperative follow‐up (x ± s, *n* = 145).

Parameters	t‐PVP (*n* = 71)	ud‐PVP (*n* = 74)	*p*
LKA	13.56 ± 6.46	13.10 ± 6.82	0.85^U^
AVH	16.05 ± 3.77	16.62 ± 3.70	0.28^U^
LL	42.59 ± 12.50	44.76 ± 13.67	0.32^T^
TLK	16.16 ± 8.62	13.80 ± 7.43	0.26^U^
Changed AVH	1.01 ± 0.77	0.57 ± 0.5	< 0.01^T^
Changed LKA	2.30 ± 1.73	1.32 ± 1.3	< 0.01^T^

*Note:* The symbol ‘U’ indicates the data were analyzed by the unpaired Mann–Whitney *U* test; The symbol ‘T’ indicates the data were analyzed by the unpaired *t* test; The symbol ‘F’ indicates the data were analyzed by the Fisher's exact test; The symbol ‘C’ indicates the data were analyzed by the Chi‐square test.

Abbreviations: t‐PVP, traditional puncture percutaneous vertebroplasty; ud‐PVP, unilateral dual‐plane puncture percutaneous vertebroplasty.

**TABLE 5:** Comparisons of postoperative VAS and ODI scores between traditional puncture PVP and unilateral dual‐plane puncture PVP (x ± s, *n* = 145).

Time	VAS			ODI		
	t‐PVP (*n* = 71)	ud‐PVP (*n* = 74)	*p*	t‐PVP (*n* = 71)	ud‐PVP (*n* = 74)	*p*
1 day after surgery	3.03 ± 0.91	2.88 ± 0.94	0.28^U^	47.31 ± 9.11	45.96 ± 9.99	0.30 ^U^
1 month after surgery	2.51 ± 1.07	2.41 ± 1.16	0.51^U^	39.62 ± 9.15	37.42 ± 10.55	0.09^U^
3 months after surgery	1.90 ± 1.19	1.80 ± 1.26	0.58 ^U^	33.03 ± 7.77	31.89 ± 9.16	0.42 ^T^
1 year after surgery	2.03 ± 0.99	1.26 ± 0.81	< 0.01 ^U^	32.45 ± 7.25	28.58 ± 7.14	< 0.01 ^U^

*Note:* The symbol ‘U’ indicates the data were analyzed by the unpaired Mann–Whitney *U* test; The symbol ‘T’ indicates the data were analyzed by the unpaired *t* test.

Abbreviations: t‐PVP, traditional puncture percutaneous vertebroplasty; ud‐PVP, unilateral dual‐plane puncture percutaneous vertebroplasty.

### 3.4 Re‐Collapse

**FIGURE 5:**
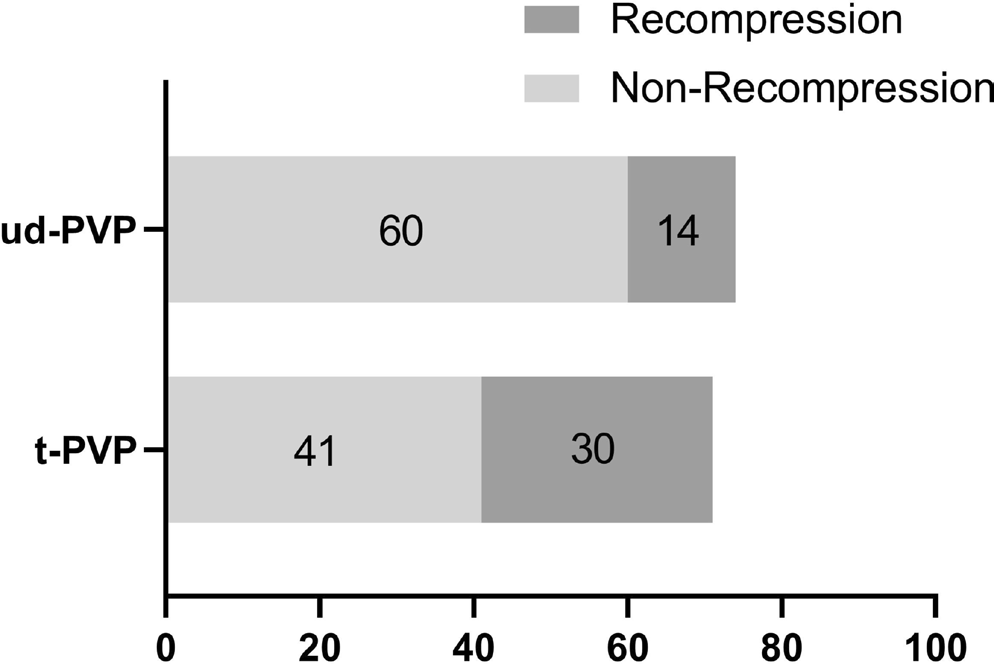
The comparison of postoperative vertebral collapse rates between the traditional group and the unilateral dual‐plane puncture group.

Subsequently, we delved into the occurrence of re‐collapse following PVP surgery. Within the traditional group, there were 30 cases (42.25%) of re‐collapse within 1 year post‐injury, significantly higher than the 14 cases (18.92%) observed in the unilateral dual‐plane group (Figure 5). This suggests that the unilateral dual‐plane puncture technique can significantly reduce the incidence of vertebral re‐collapse following PVP surgery.

Further stratification was conducted based on whether the bone cement made simultaneous contact with the superior and inferior endplates. Among those in contact with both endplates, the re‐collapse rate within 1‐year post‐injury was merely 3.9%. Conversely, the re‐collapse rate post‐surgery for cases where the bone cement only contacted one endplate or did not engage with the endplates at all was 60.29% (Table 6). This underscores the superior supportive effect provided by simultaneous contact with the superior and inferior endplates. Subsequently, we analyzed the imaging data of patients who experienced collapse despite contact with the superior and inferior endplates and found that all three cases were in the unilateral dual‐plane puncture group. Despite simultaneous contact with the superior and inferior endplates, the adjustment in the angle of the puncture needle led to the bone cement not forming a contiguous structure within the vertebral body but rather a “bone cement‐trabecular bone‐bone cement” structure resembling a sandwich biscuit. This sandwich structure may concentrate stress on the intervening trabecular bone, rendering it prone to collapse.

**TABLE 6:** Comparisons of the impact of surgical procedure and bone cement distribution on vertebral re‐collapse (x ± s, *n* = 145).

Factors	Non‐recollapse group	Re‐collapse group	*p*
Surgical procedure			
t‐PVP	41 (57.75%)	30 (42.25%)	< 0.01^F^
ud‐PVP	63 (81.08%)	14 (18.92%)	
Bone cement distribution			
Full endplate contact	74 (96.1%)	3 (3.9%)	< 0.01^F^
Partial or no endplate contact	27 (39.71%)	41 (60.29%)	

*Note:* The symbol ‘F’ indicates the data were analyzed by the Fisher's exact test.

To ascertain whether the unilateral dual‐plane puncture group reduced the occurrence of re‐collapse by influencing the distribution of bone cement, we performed binary logistic regression analysis. When the puncture method was considered as a single‐factor variable, we found a significant association between the puncture method and re‐collapse occurrence. However, upon introducing bone cement distribution into the model, the puncture method no longer served as a significant risk factor for re‐collapse. This suggests that the unilateral dual‐plane puncture may mitigate the risk of vertebral re‐collapse by altering the distribution of bone cement.

## 4 Discussion

This study demonstrated that the novel unilateral dual‐plane puncture technique for percutaneous vertebroplasty (PVP) significantly improved bone cement distribution, achieving better contact with both the superior and inferior endplates of the vertebra compared to traditional techniques. This enhanced distribution was associated with a reduction in the incidence of vertebral re‐collapse and improved long‐term clinical outcomes, including lower Visual Analog Scale (VAS) and Oswestry Disability Index (ODI) scores.

### 4.1 Prevalence and Impact of OVCFs

The incidence of osteoporosis has risen to the seventh position among common diseases worldwide [24]. In China, the current elderly population is approximately 130 million, with an annual growth rate of 3.2% for individuals aged 60 and above. The prevalence of osteoporosis in China is around 6.6%, affecting a total of 60–80 million individuals, primarily among those aged 60 and above and postmenopausal women [25, 26, 27, 28]. Within the osteoporotic population, approximately 700,000 vertebral compression fractures occur annually, constituting a significant factor leading to disability and even death in the elderly [29].

Patients with OVCFs often have multiple comorbidities, which may affect the outcomes of surgical interventions such as PVP. Studies have reported that vertebral morphology in patients with type 2 diabetes begins to deteriorate from the sixth month after PVP compared to patients without diabetes [30]. In addition, compared to patients with primary osteoporotic vertebral compression fractures, patients receiving corticosteroid treatment have a twofold increased risk of developing new fractures after PVP [31]. In this study, we excluded patients who had been on long‐term corticosteroid therapy. Moreover, there was no significant difference in the incidence of diabetes between the traditional puncture group and the unilateral dual‐plane puncture group.

### 4.2 Bone Cement Injection and Re‐Collapse Post‐PVP

After a vertebral compression fracture, the original strength and stiffness of the vertebra are compromised, especially in the collapsed trabeculae within the upper half of the vertebral body. The repair of a fractured vertebra is directly influenced by the amount of bone cement, and an appropriate dosage can directly enhance the stiffness and strength of the operated vertebra, stabilize the vertebra, correct spinal deformities, and prevent further collapse. Cadaveric studies suggest that to restore the compressive strength of a vertebra, at least 15% cement filling is required. In the thoracolumbar spine, this translates to an average injection volume of 4–6 mL of bone cement per vertebra [32]. Li et al. found that the average cement usage in patients with re‐fractures (3.30 ± 0.84 mL) was significantly lower than in those without fractures (4.46 ± 1.10 mL) [33]. Insufficient cement injection may lead to inadequate vertebral filling, insufficient support, and an increased risk of re‐collapse. Injecting more cement can allow for a wider dispersion within the trabecular bone clefts, reducing areas without cement support. Therefore, an appropriately high‐dose cement can lower the risk of further height loss post‐enhancement.

Insufficient bone cement injection is a risk factor for post‐PVP vertebral re‐collapse, and an adequate amount of bone cement is beneficial for better vertebral height restoration and stability. However, excessive bone cement injection increases the risk of cement leakage, with the potential for disastrous consequences such as nerve compression and severe pulmonary embolism [34]. Therefore, while ensuring an appropriate amount of bone cement injection, the distribution pattern of bone cement within the vertebra has become a focal point of study for some researchers.

Studies suggest that the distribution of bone cement between the upper and lower endplates of the vertebra is more effective [35]. When the bone cement fully contacts both the upper and lower endplates, it enhances the restoration of vertebral body strength and preserves vertebral body height more effectively, thereby reducing the risk of vertebral body recompression and long‐term pain. Based on this, some physicians adopt the surgical approach of bilateral dual‐plane puncture PVP, intending to achieve contact between bone cement and the upper and lower endplates. However, bilateral dual‐plane puncture PVP increases surgical time, fluoroscopy frequency, and trauma levels [36]. In our study, the average surgical duration for the unilateral dual‐plane puncture group was 38.2 min, with an average fluoroscopy frequency of 19.4 times. Zhang et al. reported that the average surgical duration for bilateral puncture PVP was 53.6 min, with a fluoroscopy frequency of 21.5 times [37]. A systematic review comparing unilateral and bilateral PVP punctures indicated that the average surgical time for unilateral puncture ranged from 20.22 to 41.2 min, while for bilateral puncture, it ranged from 40.35 to 55.7 min [38]. This suggests that the surgical duration for unilateral dual‐plane puncture is shorter than that for bilateral puncture, with a slightly lower fluoroscopy frequency.

### 4.3 Innovation of the Unilateral Dual‐Plane Puncture Technique

We propose, for the first time, a unilateral dual‐plane puncture PVP through the transpedicular route. During puncture, the puncture needle is slightly inclined towards the plane of the upper endplate, reaching the midline of the vertebra through the lateral transpedicular route. Under C‐arm guidance, bone cement is injected, and then the puncture needle is withdrawn to the posterior edge of the vertebra. The needle angle is adjusted to incline towards the plane of the lower endplate, and after reaching the midline of the vertebra, an appropriate amount of bone cement is injected. In preliminary studies, we found that unilateral dual‐plane puncture PVP significantly increases the contact rate between bone cement and the upper and lower endplates, improving the distribution pattern of bone cement within the vertebra.

As anticipated, the unilateral dual‐plane puncture technique significantly increased the proportion of simultaneous contact between bone cement and the superior and inferior endplates compared to traditional puncture techniques. Simultaneously, it markedly reduced the probability of vertebral re‐collapse postoperatively, thereby improving long‐term clinical outcomes for patients. However, this does not imply that the unilateral dual‐plane puncture technique is devoid of additional risks. We observed that excessive adjustment of the puncture angle in the unilateral dual‐plane technique could lead to the formation of a sandwich‐like structure comprising bone cement–trabecular bone–bone cement, increasing the likelihood of collapse in the intervening trabecular bone region. Moreover, in some patients from the traditional unilateral puncture group, satisfactory bone cement distribution can indeed be achieved. For these patients, the unilateral dual‐plane puncture may not be necessary. However, if the cement distribution is unsatisfactory during the procedure and the cement fails to adequately diffuse, then the unilateral dual‐plane puncture technique serves as an ideal improvement to enhance cement distribution.

## 5 Limitations and Strengths

Our study has several limitations. Firstly, we determined the sagittal distribution of bone cement through postoperative spinal x‐ray imaging. However, accurate measurements were sometimes challenging due to the obstruction caused by the bone cement. Secondly, the criteria we defined for re‐collapse, involving a height decrease exceeding 1 mm and an angle increase greater than 3°, are relatively stringent, potentially leading to false positives. Lastly, solely assessing contact between bone cement and the superior and inferior endplates as a criterion for evaluating sagittal distribution may lack precision. A more comprehensive analysis could be achieved by quantifying the contact area between the bone cement and the endplates; however, this would require follow‐up CT data, thereby increasing radiation exposure to patients during follow‐up. This study is a single‐center study. To enhance external validity, we plan to conduct further multicenter clinical research, encompassing patients of different ages, genders, ethnicities, and comorbidities to ensure the results are widely applicable. Consistent surgical techniques, postoperative care, and outcome measurements will be implemented across all centers. Additionally, long‐term follow‐ups will be conducted to assess the sustained effects of surgical interventions and understand the long‐term outcomes across diverse settings and populations.

Moreover, the purpose of this comparison is to evaluate whether unilateral dual‐plane puncture can improve the distribution of bone cement and clinical outcomes without increasing the complexity and risks associated with bilateral puncture. Therefore, this study did not explore the clinical differences between unilateral dual‐plane puncture and bilateral puncture. We plan to include a comparison between the bilateral puncture group and the unilateral dual‐plane puncture group in future studies.

To provide additional clarity, we incorporated a brief discussion of the PICO framework, outlining the population, intervention, comparison, and outcomes as part of the study's context. This study focused on patients with osteoporotic vertebral compression fractures (OVCFs), a prevalent condition among elderly individuals with osteoporosis. The novel unilateral dual‐plane puncture technique was employed to optimize bone cement distribution. By adjusting the needle's angle during the procedure, the technique aims to increase contact with both the superior and inferior endplates, improving vertebral stability and reducing re‐collapse rates. The traditional PVP technique was used as the control group. The primary comparisons included vertebral height, local kyphotic angle (LKA), lumbar lordosis (LL), thoracolumbar kyphosis (TLK), Visual Analog Scale (VAS), and Oswestry Disability Index (ODI) scores. The study demonstrated that the unilateral dual‐plane puncture approach yielded better bone cement distribution, reduced re‐collapse rates, and improved clinical outcomes compared to the traditional technique.

## 6 Conclusion

The unilateral dual‐plane puncture technique can improve the distribution of bone cement within the vertebral body, reducing the incidence of vertebral re‐collapse following PVP surgery and enhancing long‐term clinical outcomes for patients postoperatively.

## Author Contributions

Huo‐Liang Zheng and Chang‐hai Liu wrote the article. Lei‐Sheng Jiang, Chang‐hai Liu, and Huo‐Liang Zheng were responsible for data collection and analysis. Sheng‐Dan Jiang was responsible for reviewing the data. Xin‐Feng Zheng and Sheng‐Dan Jiang were responsible for reviewing and revising the article.

## Ethics Statement

The study was conducted in accordance with the ethical standards laid down in the 1964 Declaration of Helsinki and its later amendments and was approved by the Ethics Committee of Xinhua Hospital Affiliated with Shanghai Jiaotong University School of Medicine(XHEC‐QT‐2021‐086).

## Conflicts of Interest

The authors declare no conflicts of interest.

## Data Availability

Data are available from the corresponding author.
